# The Genetic Interpretation of Area under the ROC Curve in Genomic Profiling

**DOI:** 10.1371/journal.pgen.1000864

**Published:** 2010-02-26

**Authors:** Naomi R. Wray, Jian Yang, Michael E. Goddard, Peter M. Visscher

**Affiliations:** 1 Genetic Epidemiology and Queensland Statistical Genetics, Queensland Institute of Medical Research, Brisbane, Australia; 2 Department of Food and Agricultural Systems, University of Melbourne, Melbourne, Australia; 3 Victoria Department of Primary Industries, Melbourne, Australia; University of California San Diego and The Scripps Research Institute, United States of America

## Abstract

Genome-wide association studies in human populations have facilitated the creation of genomic profiles which combine the effects of many associated genetic variants to predict risk of disease. The area under the receiver operator characteristic (ROC) curve is a well established measure for determining the efficacy of tests in correctly classifying diseased and non-diseased individuals. We use quantitative genetics theory to provide insight into the genetic interpretation of the area under the ROC curve (AUC) when the test classifier is a predictor of genetic risk. Even when the proportion of genetic variance explained by the test is 100%, there is a maximum value for AUC that depends on the genetic epidemiology of the disease, i.e. either the sibling recurrence risk or heritability and disease prevalence. We derive an equation relating maximum AUC to heritability and disease prevalence. The expression can be reversed to calculate the proportion of genetic variance explained given AUC, disease prevalence, and heritability. We use published estimates of disease prevalence and sibling recurrence risk for 17 complex genetic diseases to calculate the proportion of genetic variance that a test must explain to achieve AUC = 0.75; this varied from 0.10 to 0.74. We provide a genetic interpretation of AUC for use with predictors of genetic risk based on genomic profiles. We provide a strategy to estimate proportion of genetic variance explained on the liability scale from estimates of AUC, disease prevalence, and heritability (or sibling recurrence risk) available as an online calculator.

## Author Summary

Genome-wide association studies in human populations have facilitated the creation of genomic profiles that combine the effects of many associated genetic variants to predict risk of disease. However, genomic profiles are inherently constrained in their ability to classify diseased from non-diseased individuals dictated by the genetic epidemiology of the disease. In this paper, we use a genetic interpretation to provide insight into the constraints on genomic profiles for risk prediction. We provide a strategy to estimate proportion of genetic variance explained on the liability scale from estimates of AUC, disease prevalence, and heritability available as an online calculator.

## Introduction

Genome-wide association studies in human populations have facilitated the creation of genomic profiles which combine the effects of many associated genetic variants to predict risk of disease. Genetic testing has long been available for Mendelian genetic diseases for which variants within one gene are directly responsible for the disease. In contrast, the etiology of complex genetic diseases, such those listed in Table 1, comprises both genetic and environmental risk factors. Results from genome-wide association studies have provided empirical evidence that very few associated genetic variants with effect size greater than odds ratio of 1.5 exist [1],[2]. Reconciliation of these effect sizes with the, often sizeable, estimates of heritability for many complex diseases (Table 1) means that we must expect there to be many (perhaps thousands) of genetic variants underlying complex disease if the effect size of any one variant is very small. It follows that each individual will carry a different, probably unique, portfolio of risk alleles. Whereas common risk variants have size too small to be used individually as risk predictors, profiles based on many associated genetic variants could provide useful predictions of genetic risk [3],[4]. We define genetic risk as the risk of disease given an individual's unique multi-locus genotype; genetic risk remains unchanged throughout an individual's lifetime and so could be predicted at birth prior to exposure to many environmental risk factors. Indeed, such risk predictions could be age specific, for example, risk of type 2 diabetes at 10 years, 20 years or 50 years if genomic profile sets based on empirical data were available for these scenarios which have age-specific genetic epidemiologies. As more variants are identified in the coming years, there will be increasing interest in the prospects of genomic profiling. It has been argued that genomic profiles should be assessed in terms of their clinical validity as diagnostic classifiers [5],[6]. The receiver operator characteristic (ROC) curve [7] is a well established tool for determining the efficacy of clinical diagnostic and prognostic tests in correctly classifying diseased and non-diseased individuals and has been used in the context of genomic profiling e.g., [6],[8],[9]. While the area under the ROC curve (AUC) is an important measure for clinical validity it does not tell the whole story as it does not differentiate between the accuracy with which the genomic profile predicts the true genetic risk of individuals and the accuracy with which true genetic risk predicts disease status, which is not under our control. We believe that the ability to differentiate between these components (i.e. the distinction between prediction of genotype and phenotype) is important for interpretation of the value of the genomic profile, particularly as the use of genomic profiles is very much in its infancy at present. Our knowledge of the genetic epidemiology of a disease means that we can know *a priori* that genomic profiles might not, on their own, be accurate diagnostic classifiers. For this reason, genomic profiles should judged in the first instance on the basis of their analytic validity [10] as predictors of *genetic* rather than *absolute* risk. Of course, in the long term genomic profiles can be combined with environmental risk factors to predict absolute risk in the context of clinical utility. Genomic profiles should improve upon family history which has long been used as a crude estimate of genetic risk (see Text S1).

**Table 1:** AUC related statistics for complex genetic diseases.

							*AUC* =	0.75^d^
Disease with reference for *K* and *λ_S_*	*K*×100	*λ_S_*	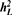	*AUC_max_* a	*AUC_half_* b	*AUC_quar_* c	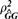	(*λ_S_*_[x]_ – 1)/(*λ_S_* – 1),
Age related macular degeneration [21],[22]	11.8	2.2	0.68	0.92	0.81	0.72	0.31	0.27
Unipolar disorder [33],[34]	10	1.7	0.39	0.84	0.74	0.67	0.52	0.49
Coronary Artery Disease [35]	5.6	3.2	0.72	0.95	0.84	0.75	0.25	0.18
Breast cancer [36]	3.6	2.5	0.44	0.89	0.79	0.71	0.36	0.29
Type-II diabetes [37]	3	3.5	0.60	0.94	0.84	0.75	0.25	0.18
Prostate cancer [36]	2.4	2.8	0.44	0.90	0.80	0.72	0.33	0.25
Asthma [38]	2	2.6	0.37	0.88	0.79	0.71	0.37	0.29
Lung cancer [36]	1.7	6.1	0.76	0.98	0.89	0.80	0.17	0.09
Colon cancer [36]	1.5	5.1	0.64	0.96	0.87	0.77	0.20	0.12
Bladder Cancer [36]	1	1.7	0.16	0.79	0.71	0.65	0.74	0.70
Stomach cancer [36]	1	6	0.63	0.97	0.88	0.78	0.19	0.10
Bipolar disorder [39]	1	6.8	0.69	0.97	0.89	0.80	0.17	0.08
Bipolar disorder [40]	0.45	7.9	0.60	0.97	0.90	0.80	0.17	0.07
Schizophrenia [25],[41]	0.85	8.6	0.76	0.98	0.90	0.81	0.15	0.07
Schizophrenia [40]	0.4	9	0.63	0.98	0.90	0.80	0.15	0.06
Rheumatoid Arthritis [42]	0.75	8	0.70	0.98	0.90	0.80	0.16	0.07
Type-I diabetes [43]	0.54	13.7	0.86	1.00	0.93	0.84	0.12	0.04
Crohn's disease [44]	0.1	26	0.76	1.00	0.95	0.86	0.10	0.02
Systemic lupus erythematosus [45]	0.03	30	0.64	1.00	0.95	0.86	0.10	0.02

**a** *AUC_max_* is the maximum AUC possible based on the genetic epidemiology parameters of disease prevalence (*K*) and sibling recurrence risk i.e. (*λ_S_*) when all the known genetic variance is explained by the genomic profile, 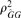 = 1.

**b** *AUC_half_* is the AUC possible if the variants included in the genomic profile explain half of the known genetic variance i.e., 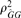 = 0.5.

**c** *AUC_quar_* is the AUC possible if the variants included in the genomic profile explain 1/4 of the known genetic variance i.e., 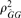 = 0.25.

**d** 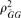 and (*λ_S_*_[x]_ – 1)/(*λ_S_* – 1), proportion of sibling risk explained, when the measured AUC for a genomic profile is 0.75.

In this paper, we provide insight into the genetic interpretation of AUC. We begin by considering quantitative traits for which the concepts of accuracy of risk prediction are well developed. For disease traits we differentiate between measures on the observed scale of disease versus the underlying scale of disease risk as we believe recognition of scale of measurement is often overlooked. We define *AUC_max_* as the maximum AUC that could be achieved for a disease when the test classifier is a perfect predictor of genetic risk. We quantify the relationship between *AUC_max_* and heritability of liability and disease prevalence (lifetime morbidity risk). We show how to interpret AUC (which is a measure on the observed disease scale) of a genomic profile as the proportion of variance explained (or accuracy of prediction squared) on the underlying liability scale. Finally, we benchmark the value of genomic profiles by comparing them to the AUC expected when family history resulting from shared genetic risk factors is used as a predictor of genetic risk.

## Methods

### Background: quantitative traits

For quantitative traits, in which phenotypic scores are (or can be transformed to be) normally distributed, the efficacy of a genomic profile is naturally expressed as the proportion of the genetic variance explained by the profile. The variance in phenotypes, *V_P_*, can be partitioned into variance of genetic values, *V_G_*, so that the proportion of the variance that is genetic is the heritability *V_G_/V_P_*. Genomic profiling provides a direct estimate, 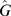, of true genetic values, *G*, for individuals in a population and the efficacy of a genomic profile can be expressed as the proportion of the genetic variance explained by the profile 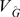/*V_G_*. We define 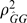 = 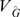/*V_G_*, since in selection theory [11], used in livestock and plant breeding, the correlation between predicted and true genetic risk (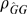) is used as the measure of accuracy of prediction, 
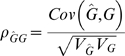
, and if the predictor is unbiased (the regression of *G* on 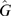 is 1), 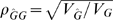. The ratio 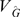 */V_P_* is estimated as the *R* ^2^ from the regression of *P* on 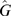 and is interpreted recognising its upper limit to be *V_G_/V_P_* or heritability. These measures show that for quantitative traits, the accuracy with which the genomic profile predicts genetic risk is clearly separable from accuracy with which the true genetic risk predicts the phenotype. In contrast, AUC is a measure of the efficacy with which 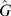 predicts phenotype which, as shown below, has an upper limit constrained by the heritability, and also prevalence, of the disease.

### Background: disease traits

For disease traits, the phenotype has two possible values, either affected or not affected. On this observed scale, the directly measurable genetic parameters are those of recurrence risks to relatives, *λ_R_* for relatives of type *R*, which is the ratio of the prevalence of disease in the relatives of affected individuals (*K_R_*) compared to the prevalence in the population (*K*),
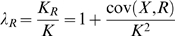
where cov(*X*, *R*) the covariance in disease status between diseased individuals *X* and their relatives on the observed disease risk scale [12]. For example, when the relatives are monozygous twins (*R* = *MZ*), Cov(*X*,*MZ*) = 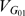 the genetic variance, with the subscript “01” denoting the all-or-none disease risk scale. On this scale, the majority of the genetic variance is non-additive, especially when disease prevalence is low [13],[14]. The broad sense heritability on this scale is 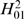 = (*λ_MZ_* -1)*K*/(1-*K*) where *λ_MZ_* is the monozygotic twin recurrence risk, assuming there is no common environmental component to the recurrence risk. 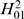 is not a normally reported statistic because of its dependence on disease prevalence [15]. If the relatives are siblings (*R* = *S*) then *λ_S_* is the sibling risk ratio and Cov(*X*,*S*) = 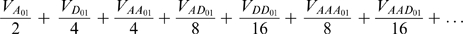 [11], where the variance subscripts *A* and *D* denote additive and dominance terms, and in combination denote epistatic variance terms. Thus, although *λ_S_* is an estimable quantity, it is not simply related to the genetic variances on the observed binary scale.

The genetic properties of disease are much more easily understood by using the threshold liability model [11], in which risk of disease is transformed to a normally distributed liability scale *P ∼N*(0, 1) and *P* = *A* + *E*, where *A*∼*N*(0, 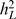) are the genetic effects on the liability scale. On this scale the genetic effects combine in an additive way; 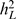 is the narrow sense heritability on the liability scale (or heritability of liability) and on this scale broad sense and narrow sense heritability are equal. *E* are independent environmental effects, *E∼N*(0,1-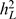). The biological plausibility of an underlying normally distributed liability to disease is based on the assumption that complex traits are influenced by many variables; the central limit theorem states that the distribution of the sum of independent random variables approaches normality as the number of variables increases. Under the threshold liability model individuals are affected when *P* >*T*, where *T* is the threshold on the normal distribution which truncates the proportion of affected individuals or disease prevalence (i.e., *K*), *T* = Φ^−1^(1-*K*), Φ(*T*) = 1-*K*, where Φ(*T*) is the cumulative density function of the normal distribution up to values of *T*, e.g. if *K* = 0.05, *T* = 1.645. The threshold liability of risk scale has much nicer properties than the observed disease scale and provides a framework for comparison of scenarios independent of disease prevalence. The relationship between heritability of liability 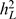 and the directly estimable parameters of *K* and *λ_S_ is*

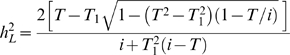
(1)
[16] with 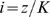 and *z* the height of the standard normal curve and *T*_1_ = Φ^−1^(1- *λ_S_ K*), i.e. the threshold *T*_1_<*T* when *λ_S_*>1, reflecting that the prevalence amongst sibs of affected individuals, *K_S_* is greater than the prevalence in the population as a whole (e.g. if K = 0.05 and *λ_S_* = 2, *z* = 0.103, *T*_1_ = 1.282, 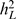 = 0.371).

### Area under the ROC curve

The AUC is a statistic calculated on the observed disease scale and is a measure of the efficacy of prediction of phenotype using a test classifier. The ROC plots the true positive rate (TPR or sensitivity) against the false-positive rate (FPR or 1-specificity). TPR  = probability (positive test result|diseased) and FPR  =  probability (positive test result|not diseased). Since these probabilities are conditional, they are not dependent on the number of cases or controls tested, except through the sampling variance associated with them. In genomic profiling the ROC is obtained by ranking a set of individuals with known disease status by their genomic profile from lowest estimated risk (i.e., profile score) to highest estimated risk and then assessing sensitivity and specificity assuming a cut-off after each rank (starting with the highest ranked individual). If *n_d_* and *n_d'_* are the numbers of diseased and not diseased individuals, and if the individual with the highest predicted genetic risk has rank *r_1_* = *n_d_* + *n_d'_* = *n*, AUC can be calculated directly from the mean rank of the diseased individuals (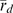),
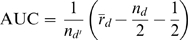
(2)(see example in Figure S1). Equally, AUC can be calculated as AUC = 0.5(1 + *D*) where *D* is the Somers' rank correlation [17] between risk profile and disease status (1 =  diseased, 0 =  not diseased). Another equivalent definition of AUC is the probability that a randomly selected pair of diseased (*d*) and non-diseased (*d'*) individuals are accurately classified [18]. The probability is the same as the probability that difference between the genetic liability of the *d* and *d'* individuals is greater than zero. This difference is approximately normally distributed with mean *μ_d_* - *μ_d'_* and variance 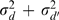. Using the liability threshold model and results of standard genetic selection theory [11] the means (*μ*) and variances (*σ* ^2^) of the genetic liability of *d* and *d*' individuals are
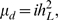


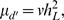


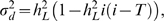


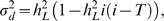
where *v* = -*iK/*(1 – *K*). The genetic liabilities of the *d* and *d'* groups are each approximately normally distributed, the approximation being less accurate for high heritabilities.

Therefore,
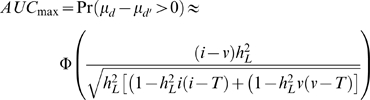
(3)


### Using AUC measured by a genomic profile to estimate the proportion of genetic variance explained

A useful property of AUC (as discussed above) is that for a given disease the estimated AUC is independent of the relative proportions of cases and controls in the sample being classified [7], i.e. the mean rank is approximately the same if the proportion of cases: controls is *K*: (1-*K*) or 1∶1. Or equally, the probability of a randomly selected case and control being correctly ranked is independent (except for sampling) of the number of cases and controls measured. We can use equation 3 to estimate the variance on the liability scale explained by a genomic profile, *x*, by making 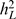 the subject of the equation, but renaming it as 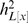, recognising that it represents the proportion of variance explained by the profile. Then, from two measurable parameters, *K* and *AUC*, we can calculate 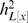,
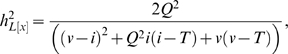
(4)


Where *Q* = Φ^−1^(*AUC*). From this, we can calculate the proportion of the known genetic variance explained by the genomic profile
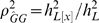
(5)using the estimates of *K* and *λ_S_* to calculate 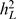 (equation 1). We can also calculate the proportion of the sibling risk explained by the profile, (*λ_S_*_[x]_ – 1)/(*λ_S_* – 1), where *λ_S_*_[x]_ = (1-Φ(*T*_1[*x*]_))/*K* and
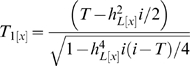
(6)
[19]. 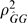 and (*λ_S_*_[x]_ – 1)/(*λ_S_* – 1) measure the same concept but in different ways and on different scales; both are useful criteria for assessing the extent to which the genomic profile accounts for the known genetic component of disease. We consider family history as a predictor of genetic risk in the Text S1.

### Simulation

We used simulation under the liability threshold model [11],[14] to check our derivations. We simulated 100,000 nuclear families sampling risk on the liability scale, *P* = *A* + *E*, *A ∼ N*(0, 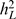) for parents, and *A* = ½*A_dad_*+½*A_mum_*+*A_mend_* for children, where the Mendelian segregation terms were random numbers sampled as *A_mend_* ∼ *N*(0, ½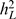); E ∼ *N*(0,1 - 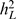). Individuals were considered affected, *P*_01_ = 1, if *P >*Φ^−1^(1-*K*) = *T*, otherwise individuals were not affected and *P*_01_ = 0. Genetic values on the observed scale, *G_01_*, were calculated as the normal probability, 
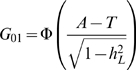
. From this we could calculate 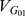, 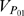, 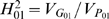
(using the *G_01_* and *P_01_* of the first child from each family) and sibling recurrence risk. *AUC_max_* was calculated from the mean rank of diseased individuals using equation 2 when ranked on *A*.

## Results

### The maximum value of AUC when the test classifier is a genetic predictor depends on heritability and disease prevalence

**Figure 1:**
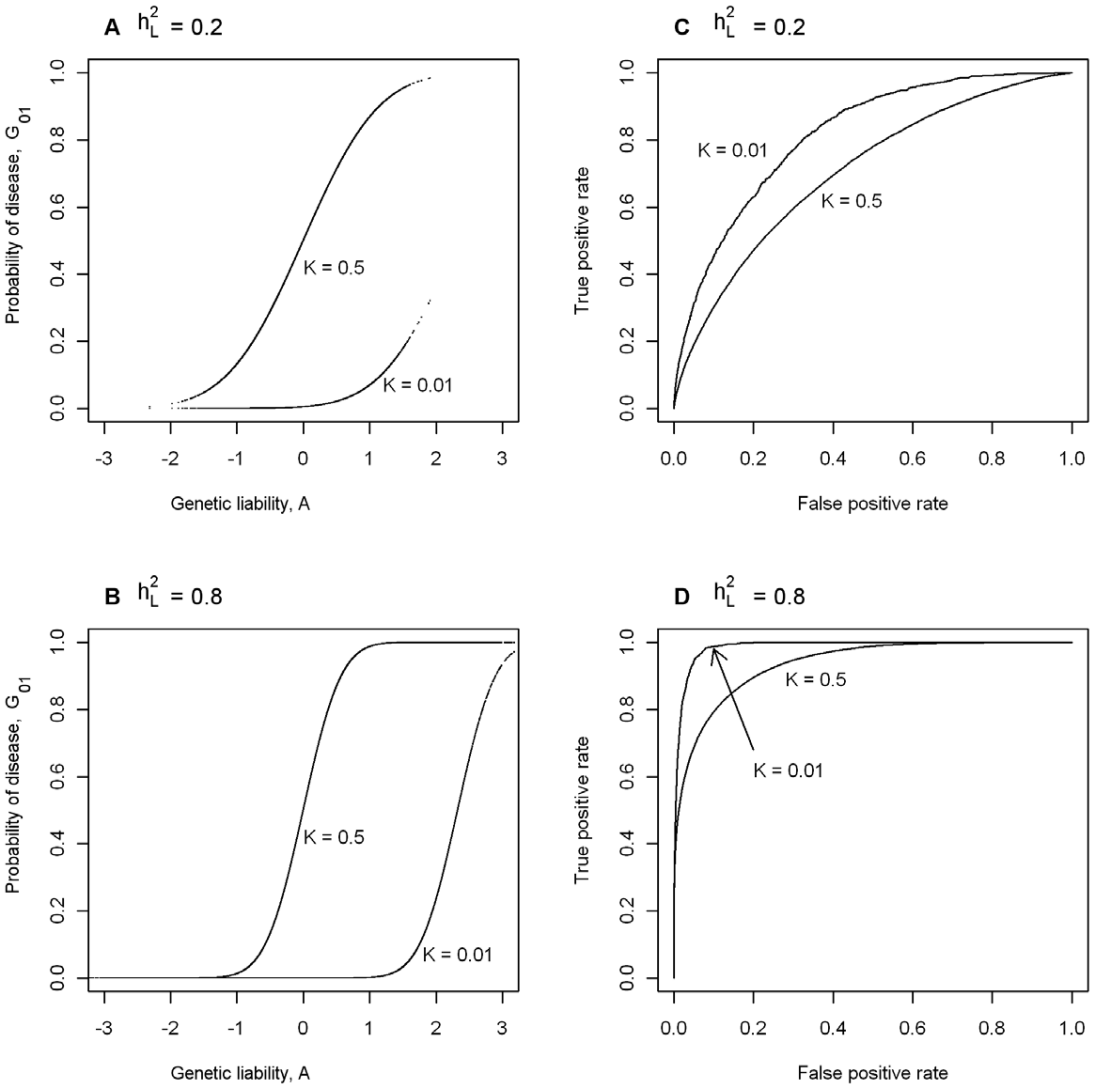
The dependence of maximum AUC (*AUC_max_*) from a genomic profile on heritability and disese prevalence. (A,B) Probability of disease versus genetic liability. (C,D) ROC curve [46].

In Figure 1A we consider two diseases both with heritability of liability, 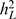 = 0.2, plotting probability of disease (*i.e. G_01_*) vs genetic liability (*i.e. A*). To allow an extreme comparison, one of the diseases has prevalence *K* = 0.5 and the other, *K* = 0.01. Figure 1B also considers two diseases with prevalences *K* = 0.5 and 0.01, but in this case both have 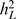 = 0.8. In Figure 1A and 1B, the position of the rise in probability of disease along the x-axis reflects the disease prevalence and the steepness of the rise reflects the heritability of the disease. In Figure 1A the distribution of genetic liabilities on the underlying scale is exactly the same for these two diseases, but when *K* = 0.01 higher genetic liabilities are needed before probability of disease rises above virtual zero (virtual because it is not exactly zero, but very close to zero); similarly for the diseases in Figure 1B. Figure 1C and 1D plot the ROC curves for the diseases considered in Figure 1A and 1B, respectively. These graphs demonstrate firstly (not unexpectedly), that for diseases with the same prevalence, genetic liability is a better predictor of disease status for diseases with higher heritability and secondly, that for diseases with the same heritability, genetic liability is a better predictor of disease status for rarer diseases, because a higher proportion of those with high genetic liability are actually diseased. For example, if we used genetic liability of ≥1 as our predictor of disease, then the TPR is 0.26 and the FPR = 0.00, when *K* = 0.5, compared to TPR = 0.99 and the FPR = 0.12, when *K* = 0.01. These graphs demonstrate that maximum value of AUC (i.e. *AUC_max_*) when the test classifier is a genetic predictor is dependent on both 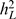 and *K*.

### Prediction of *AUC_max_* from 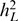 (or *λ_S_*) and *K*

**Figure 2:**
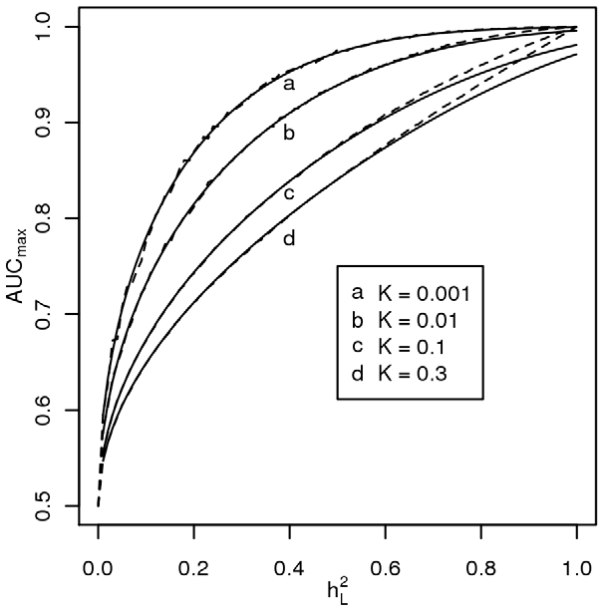
Relationship between maximum AUC (*AUC_max_*) from a genomic profile and heritability on the liability scale 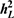. For different disease prevalences (A–D) from simulation (dashed line) and from equation 3 (solid line).

**Figure 3:**
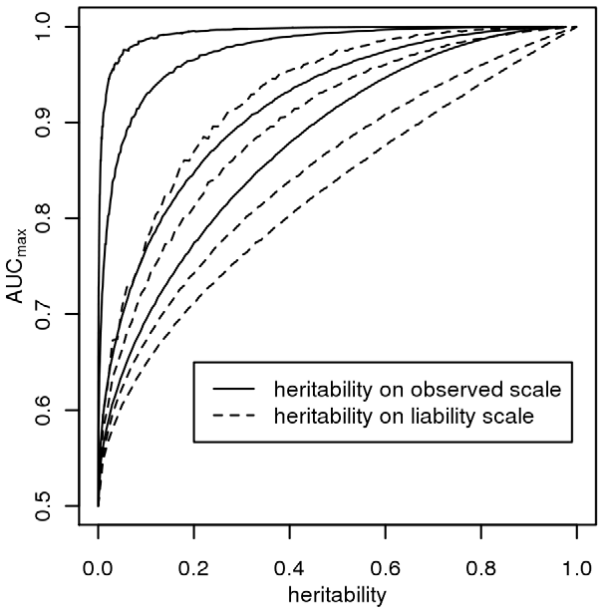
The relationship between maximum AUC (*AUC_max_*) from a genomic profile and heritability on the liability scale 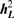 (dashed line) or heritability on the observed scale 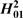 (solid line), for disease prevalences in order from top left, *K* = 0.001, 0.01, 0.1, 0.3.

Figure 2 plots *AUC_max_* vs 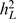, for *K* = 0.001, 0.01, 0.1, 0.3 from simulation (dashed line) and from equation 3 (solid line) and shows that *AUC_max_* is particularly constrained for more common or low heritability diseases. Jannsens et al [3], in their Fig. 4, have shown the relationship between AUC and the proportion of variance on the disease scale explained by the genomic profile; since their genomic profile assumed all genetic variants were known without error their graph represents the relationship between *AUC_max_* and 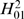. Our simulation results provided the same relationship when plotted on this scale (Figure 3, solid line). In Figure 3 we show the relationship of *AUC_max_* with 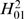 and 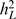 (for each simulation combination of *K* and 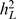, the *AUC_max_* and 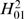 are calculated).

### Complex genetic diseases

Table 1 lists *AUC_max_* for a range of complex genetic diseases calculated using equation 3, with 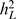 calculated using equation 1 from published estimates of *K* and *λ_S_*. Despite being observable, the parameters *K* and *λ_S_* are subject to considerable sampling variance; we have tried, where possible, to take estimates from reviews or large studies, but large study samples simply do not exist for some low prevalence disorders. The values of *AUC_max_* show that it should be possible for a genomic profile for complex diseases to exceed 0.75, the threshold regarded [20] as making a diagnostic classifier clinically useful when applied to a sample considered to be at increased risk. However, based on the results in Table 1 only the diseases with high heritability and low prevalence, such as Type I diabetes, Crohn's Disease and Lupus, can achieve an AUC, by genomic profiling alone, above the 0.99 threshold regarded [20] as being required for a diagnostic classifier to be applied in the general population. In Table 1, we also consider the AUC expected under scenarios where a genomic profile accounts for only a half (*AUC_half_*) or a quarter (*AUC_quar_*) of the known genetic variance. These results show that for rare diseases genomic profiles can be useful classifiers of disease (*AUC*>0.8 when *K*<0.01), when the profile explains only a quarter of the genetic variance.

Using equations (4) and (5) we calculate 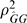 for the diseases listed in Table 1 when AUC = 0.75. The results (Table 1) show that the same AUC can represent quite different successes of the genomic profile in representing the known genetic variance, ranging from 0.10 to 0.74. If we are able to explain half of the known genetic variance with identified risk variants then genomic profiles for most complex genetic disease (*AUC_half_,* Table 1) will achieve some clinical validity as AUC is >0.75 for all but bladder cancer, for the examples provided.

### Example: age related macular degeneration

Consider the first listed example in Table 1, age related macular degeneration (AMD).

Based on the review of Scholl et al [21] and the large twin study of Seddon et al [22] we have used a prevalence after 80 years age of advanced AMD *K* = 11.8% and a sibling recurrence risk representing the genetic contribution of *λ_S_* = 2.2, which correspond to heritability on the liability scale of 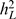 = 0.68 (equation 1). If the genetic test explains all the genetic variance (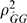 = 1), the maximum AUC that could be achieved by a genomic profile is *AUC_max_* = 0.92. If only half or a quarter of the genetic variance can be detected by genomic markers then the maximum AUC that can achieved are *AUC_half_* = 0.81 and *AUC_quar_* = 0.72, respectively, values that exceed the prediction of genetic risk based of the most optimistic scenario from a prediction based on family history (Text S1). If complete disease status is known for all siblings, parents, grandparents, aunts, uncles and cousins then the maximum AUC that could be achieved is 0.71, translating to a genomic profile that explains 0.21 of the genetic variance (Table S1). In practice, the AUC for a risk predictor based on rs1061170 a single nucleotide polymorphism in the complement factor H (*CFH*) gene was 0.69 [23] (and was approximately equal for advanced AMD cases vs controls and all AMD cases vs controls). From equations 4–6, 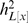 = 0.12, *λ_S_*_[*x*]_ = 1.17, 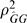 = 0.17 and (*λ_S_*_[x]_ – 1)/(*λ_S_* – 1) = 0.15.

## Discussion

### Relationship of *AUC_max_* to heritability and disease prevalence when the disease classifier is a genetic risk predictor

The AUC is a widely used statistic that summarises the clinical validity of a diagnostic or prognostic test. However, the AUC statistic of a genomic profile alone has an upper limit (*i.e. AUC_max_*) which depends on the genetic epidemiology of the disease, namely the disease prevalence and heritability. It is important that in the first instance, particularly when genomic profiling is in its infancy, that genomic profiles are judged on their ability to predict genetic risk (their analytic validity) rather than on the basis of clinical validity [10]. Since AUC is estimated as a function of a rank correlation its genetic interpretation is not immediately obvious. Here we provide a genetic interpretation of the AUC expressed in terms of it genetic epidemiology parameters (equation 3). A relationship between *AUC_max_* and heritability was first demonstrated graphically by Janssens et al [3] (see solid line Figure 3). However, their representation was of broad sense heritability on the observed scale (i.e. 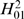) which is a little used measure of heritability because of its dependence on disease prevalence [13]. Here we show (Figure 2 and equation 3) the relationship between *AUC_max_* and the more commonly used measure of heritability, the heritability of liability (i.e., 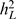) We show that *AUC_max_* is dependent on both 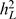 and disease prevalence (*i.e. K*).

Initially, it may seem counter-intuitive that AUC depends on disease prevalence since for an individual disease TPR and FPR are independent of the proportion of cases and controls measured and therefore of the sample prevalence. However, as we have clearly shown (Figure 1A and 1B) the dependence on disease prevalence results from our ability to generalise across diseases in the context of a test classifier being a genomic profile.

In contrast to our results and those of Janssens et al [3], Clayton [24] provided an expression for ROC under a polygenic model which is independent of population disease prevalence. His derivation assumes that the effect of each locus is additive on the log risk scale [25]. Slatkin [26] and we [27] have found that this model allows probabilities of disease that exceed one, which although they occur with low frequency can have substantial impact on the estimates of recurrence risk and genetic variance. Under this model there is a relationship between recurrence risk to monozygotic twins and to siblings of *λ_MZ_*/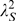 = 1; this ratio is not achieved when probabilities of disease are constrained to their natural parameter space of a maximum of 1. Furthermore, empirical estimates of the ratio of *λ_MZ_*/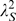 from the studies listed in Table 1 that provide estimates of *λ_MZ_* and *λ_S_* are mostly less than 1.0 [27], particularly for low prevalence diseases. Recognising that these estimates are subject to sampling variance, the estimates of *λ_MZ_*/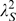 are 1.1 (AMD), 0.4 (coronary artery disease), 0.8 (breast cancer), 0.7 (schizophrenia [25]), 0.9 (rheumatoid arthritis) and 0.4 (Type I diabetes). Therefore, we believe the model used by Clayton to derive the relationship between AUC and heritability (or sibling recurrence risk) independent of disease prevalence is not valid.

### AUC and accuracy of genetic profiles

AUC is a useful measure because of its independence of the numbers of diseased and diseased individuals tested, but we advocate the reporting of an estimate of the proportion of the known genetic variance on the liability scale (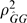) or the proportion of sibling risk accounted for by the profile and we provide a method to do this using the estimated AUC, disease prevalence and heritability on the liability scale or sibling recurrence risk (equation 5). An AUC of 0.75 can imply anything from 0.10 to 0.74 of the genetic variance explained by the genomic profile for the complex diseases listed in Table 1. The correlation 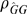 has long been the benchmark in non-human genetics of accuracy of genetic risk predictors. 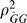 can be calculated from three measurable statistics, disease prevalence, sibling recurrence risk and AUC of the profile (using equations (1) and (4)). In this way, estimates of AUC can provide direct estimates of the proportion of ‘missing heritability’ [28] which takes into account the interdependence of identified associated variants.

Currently, the derivation of genomic profiles is very much in its infancy. As the sample size of genome-wide association studies increase, we can expect genomic profiles to include more and more validated associated variants. However, 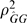 is constrained by the variance that could be detected by the markers that are genotyped recognising that the current generation of genome-wide chips explain at most ∼80% of the known variance in single nucleotide polymorphisms across the Caucasian genome [29]. This, in turn, may only be a fraction of the total genomic variance once structural variants such as copy number variants are included [30]. The actual variance explained by the profile depends on the sample size (i.e., power) of the studies from which associated genetic variants have been detected. It is likely that there are many variants which have such a small effect size that they will be impossible to detect even with very large samples. Although each such variant makes only a very small contribution to the genetic variance, there may be so many that a sizeable proportion of the variance will go undetected. Even if only quarter of the genetic variance is detectable by our future genotyping technology, the AUC is still greater for the genomic profile than for family history (ignoring shared environmental risks of family members, Text S1).

### Limitations

In our derivations we have assumed the liability threshold model [11],[14]. Slatkin [26] demonstrated that the threshold model was one of several genetic models that provided the necessary steep increase in probability of disease with increasing load of genetic risk alleles [26]. The main assumption of the liability threshold model is that the distribution of liability scores is unimodal which should be achieved as long as there is no single unidentified genetic or environmental of very large effect [11]. The model accommodates any distribution of risk allele effect sizes and risk allele frequencies as long as there are sufficient (“more than one or a few” [11]) risk alleles in the population to create an approximately normal distribution of genetic liability scores. Since our simulation results of *AUC_max_* vs 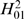 (Figure 3) based on the liability threshold model agree with those of Janssens et al [3] who used a logit model to combine genetic risks from individual genetic variants, it is clear that the dependence of *AUC_max_* on heritability and disease prevalence is not a function of the threshold model.

We have also assumed that a genetic profile is applied in the same “average” environment as the genetic risks were estimated and we have assumed that all familiality is of genetic origin. The *AUC_max_* will be lower than those derived here if any part of the sibling recurrence risk reflects co-variation of non-genetic origin. Using recurrence risks from different types of relatives, the importance of common environmental factors can be assessed and a *λ_S_* which reflects the genetic contribution of sibling recurrence can be used in our calculations. We have also assumed that the genomic profile consists of genetic markers associated with disease that are passed on according to the rules of Mendelian inheritance. In the future, a genomic profile might include non-heritable genetic variants, for example recurrent *de novo* copy number variants or perhaps methylation status variants (for which the inheritance pattern, if any, is currently unclear [31]). Such variants, although genetic, do not contribute to the similarity between relatives, and so would be included in the environmental component when partitioning variance. Under these circumstances it is possible that a genomic profile could exceed the *AUC_max_* based on sibling recurrence ratio. Our calculations assume that we know the population parameters *K* and *λ_S_* (and therefore 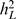). Estimates of these parameters are sometimes based on small sample size and are subject to sampling bias or different definitions of the disease. In particular, prevalence rates can depend on the age distribution of the population in which they are measured. In addition, recurrence risk ratios of relatives have a maximum possible value which is dependent on the disease prevalence, so that higher risk ratios are achievable when disease prevalence is lower; and estimates of sibling risk ratio and disease prevalence calculated in different studies sometimes reflect this dependence. In Table 1, we included two different estimates for both schizophrenia and bipolar disorder, but for these examples the estimates of *AUC_max_* are robust to the magnitude of differences reported in genetic epidemiology parameters for individual diseases. At present, genomic profiles based on validated associated variants do not come anywhere close to the maximum implied by their *AUC_max_*; Jakobsdottir et al [6] have reported AUC of 0.80 for risk of cardiovascular events, 0.64 for type 2 diabetes, 0.56 for prostate cancer, 0.66 for Crohn's Disease and 0.79 for age related macular degeneration. This is not surprising given the effect size of individual associated variants discovered in genome-wide association studies, which imply that much larger sample sizes will be needed to discover the majority of the variants that explain the genetic variance [4]. However, already these genomic profiles outperform family history (resulting from shared genetic risk only) for four out of five of these diseases. Although the AUC is a useful summary statistic for clinical validity, in practice clinical utility depends on many other factors such as the benefits versus risks of the intervention strategies that follow from the risk prediction [5],[32]; these important factors are not considered here.

### Conclusion

We have provided a genetic interpretation of and insight into the AUC statistic calculated under a genomic profile. Time will tell if genetic variants amenable to genotyping are able to reconstruct the known genetic variance in its totality. Even if it is possible to explain only a quarter of the known genetic variance, the genomic profile will be a more useful predictor of genetic risk than self-reported family history (in the absence of shared environmental risk factors) which is a commonly used measure for targeted screening programmes for complex genetic diseases. In practice, predictions of risk to disease will incorporate both genetic and environmental risk factors to produce the best predictions of absolute risk to disease. Here we provide a benchmark for the expected contribution from the genetic component of the prediction illustrating that the same AUC estimated for different diseases can imply quite different proportions of genetic variance explained by the genomic profile, which is often overlooked (e.g. [5]). Ultimately, genomic profiles may be used without contributions from environmental risk factors, since the contribution from the genomic profile can be estimated perinatally, prior to exposure by many environmental risk factors and when limited family history of disease is available. Indeed, one purpose of a genetic risk predictor is to allow individuals to choose to modify their exposure to environmental risks. We provide a simple online calculator (http://gump.qimr.edu.au/genroc) to calculate i) the maximum AUC for a genomic profile of a disease given estimates of disease prevalence and sibling recurrence risk or heritability of liability, ii) the proportion of variance explained on the liability scale given an estimate of AUC from a risk predictor and disease prevalence and iii) proportion of genetic variance or of sibling risk explained given an estimate AUC, disease prevalence and sibling recurrence risk [2].

## Supporting Information

Figure S1Example calculation of ROC curve for a genomic profile. An example of *n_d_* = 9 diseased (case) and *n_d'_* = 10 non-diseased (control) individuals listed in rank order on a genomic profile. The area under the curve is calculated from equation 2, which is derived as the sum of the horizontal rectangles (bounded by dashed lines) of the ROC plot (solid line) generated by progressing through the ranked list of individuals: each time the next ranked individual is not diseased, the ROC line moves along the *x*-axis by 1/*n_d'_* and each time the next ranked individual is diseased the ROC line moves up *y*-axis by 1/*n_d_*. The mean rank value (*r_i_*) of the cases is 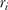 = 13.2 and AUC = 0.82.(0.07 MB TIF)

Table S1 AUC related statistics for complex genetic diseases: Table 1 with added columns considering family history.
(0.10 MB PDF)

Text S1 AUC based on family history as a prediction of genetic risk.
(0.07 MB PDF)
