# Supplementary figures and images for: The Genetic Interpretation of Area under the ROC Curve in Genomic Profiling
Source: PLoS Genet. 2010 Feb 26;6(2):e1000864. doi: 10.1371/journal.pgen.1000864 (PMC2829056; doi:10.1371/journal.pgen.1000864)

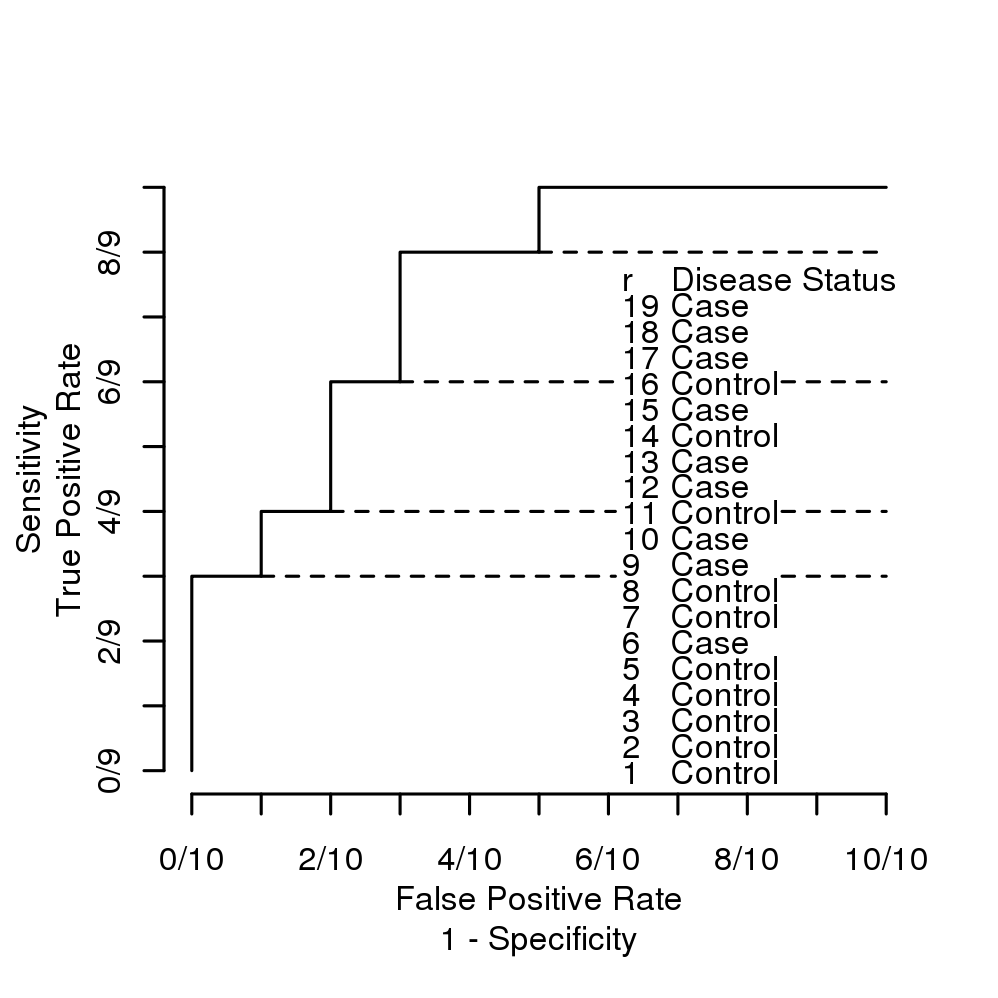

Supplement: Figure S1 — Example calculation of ROC curve for a genomic profile. An example of nd = 9 diseased (case) and nd' = 10 non-diseased (control) individuals listed in rank order on a genomic profile. The area under the curve is calculated from equation 2, which is derived as the sum of the horizontal rectangles (bounded by dashed lines) of the ROC plot (solid line) generated by progressing through the ranked list of individuals: each time the next ranked individual is not diseased, the ROC line moves along the x-axis by 1/nd' and each time the next ranked individual is diseased the ROC line moves up y-axis by 1/nd. The mean rank value (ri) of the cases is = 13.2 and AUC = 0.82. (0.07 MB TIF) [file pgen.1000864.s001.tif]
