# Supplementary material for: The Genetic Interpretation of Area under the ROC Curve in Genomic Profiling
Source: PLoS Genet. 2010 Feb 26;6(2):e1000864. doi: 10.1371/journal.pgen.1000864 (PMC2829056; doi:10.1371/journal.pgen.1000864)
Supplement: Table S1 — AUC related statistics for complex genetic diseases: Table 1 with added columns considering family history. (0.10 MB PDF) [file pgen.1000864.s002.pdf]

Table S1 AUC related statistics for complex genetic diseases: Table 1 with added columns considering family history.

Disease<br>References for K and λ_S are provided in the main text.	K×100	λ_S	h_L^2	AUC_{max} [a]	AUC_{half} [b]	AUC_{quar} [c]	AUC = 0.75[d]<br>$\rho_{\hat{G}G}^2$	$\frac{(\lambda_{S[x]}-1)}{(\lambda_S-1)}$	Full knowledge of 3 generation family history[e]<br>AUC	$\rho_{\hat{G}G}^2$
Age related macular degeneration	11.8	2.2	0.68	0.92	0.81	0.72	0.31	0.27	0.71	0.21
Unipolar disorder	10	1.7	0.39	0.84	0.74	0.67	0.52	0.49	0.62	0.12
Coronary Artery Disease	5.6	3.2	0.72	0.95	0.84	0.75	0.25	0.18	0.71	0.16
Breast cancer	3.6	2.5	0.44	0.89	0.79	0.71	0.36	0.29	0.62	0.08
Type-II diabetes	3	3.5	0.60	0.94	0.84	0.75	0.25	0.18	0.66	0.10
Prostate cancer	2.4	2.8	0.44	0.90	0.80	0.72	0.33	0.25	0.59	0.04
Asthma	2	2.6	0.37	0.88	0.79	0.71	0.37	0.29	0.58	0.04
Lung cancer	1.7	6.1	0.76	0.98	0.89	0.80	0.17	0.09	0.68	0.08
Colon cancer	1.5	5.1	0.64	0.96	0.87	0.77	0.20	0.12	0.64	0.06
Bladder Cancer	1	1.7	0.16	0.79	0.71	0.65	0.74	0.70	0.52	0.00
Stomach cancer	1	6	0.63	0.97	0.88	0.78	0.19	0.10	0.62	0.04
Bipolar disorder	1	6.8	0.69	0.97	0.89	0.80	0.17	0.08	0.63	0.05
Bipolar disorder	0.45	7.9	0.60	0.97	0.90	0.80	0.17	0.07	0.57	0.01
Schizophrenia	0.85	8.6	0.76	0.98	0.90	0.81	0.15	0.07	0.66	0.06
Schizophrenia	0.4	9	0.63	0.98	0.90	0.80	0.15	0.06	0.56	0.01
Rheumatoid Arthritis	0.75	8	0.70	0.98	0.90	0.80	0.16	0.07	0.60	0.02
Type-I diabetes	0.54	13.7	0.86	1.00	0.93	0.84	0.12	0.04	0.64	0.03
Crohn's disease	0.1	26	0.76	1.00	0.95	0.86	0.10	0.02	0.58	0.01
Systemic lupus erythematosus]	0.03	30	0.64	1.00	0.95	0.86	0.10	0.02	0.50	0.00

a: AUC_{max} is the maximum AUC possible based on the genetic epidemiology parameters of disease prevalence (K) and sibling recurrence risk (λ_S) i.e. when all the known genetic variance is explained by the genomic profile, $\rho_{\hat{G}G}^2 = 1$. **b:** AUC_{half} is the AUC possible if the variants included in the genomic profile explain half of the known genetic variance i.e., $\rho_{\hat{G}G}^2 = 0.5$. **c**: AUC_{quar} is the AUC possible if the variants included in the genomic profile explain 1/4 of the known genetic variance i.e., $\rho_{\hat{G}G}^2 = 0.25$ **d:** $\rho_{\hat{G}G}^2$ and $\lambda_{S[x]}/\lambda_S$ when the measured AUC for a genomic profile is 0.75.**e:** AUC, $\rho_{\hat{G}G}^2$ and proportion of sibling risk explained $\frac{(\lambda_{S[x]}-1)}{(\lambda_S-1)}$ by a prediction based on full knowledge of disease status of three generations of relatives from simulation . NB The online calculator presents family history results from equation S1.
