# Supplementary material for: The Genetic Interpretation of Area under the ROC Curve in Genomic Profiling
Source: PLoS Genet. 2010 Feb 26;6(2):e1000864. doi: 10.1371/journal.pgen.1000864 (PMC2829056; doi:10.1371/journal.pgen.1000864)
Supplement: Text S1 — AUC based on family history as a prediction of genetic risk. (0.07 MB PDF) [file pgen.1000864.s003.pdf]

Text S1

Family history a predictor of genetic risk

Family history is routinely recorded for diseases such as heart disease, cancer and psychiatric disorders to enable clinicians to improve diagnosis and is a recognised and promoted criterion for self-presentation of patients at screening clinics [1]. For some diseases, family history reflects risk factors of both shared genetic and common environment etiology, but for most complex genetic diseases the genetic component is more important, even for diseases with known major environmental risk factors such as coronary artery disease [2]. Family history is cheap to record, but its value is limited by small family size and personal knowledge or memory. Despite this, its recognised value is reflected in the continuing proposal of methods [3] which aim to achieve the most accurate estimate of genetic risk based of family history. But the value of family history records is inherently limited, since low disease prevalence implies low frequency of positive family history even for diseases with high heritability [4,5,6] Family history has a high positive predictive value, but a low negative predictive value [5]; i.e., a positive family history can imply a high genetic liability to disease, but negative family history implies very little about genetic liability to disease. A recent survey of the national databases of Sweden, reported a disease prevalence of 0.4% and sibling recurrence risk of 9 for schizophrenia translating to a heritability on the liability scale of 0.64. Despite this sizeable heritability, 96% of cases had no recorded family history. Indeed, even under idealised circumstances of full knowledge of the family history across three generations, and average Poisson family size of two children per couple the expected proportion of "sporadic" cases is still as high as 85% [4,6]. However, the major limitation of family history is that it can only provide the same predicted risk for all children in a nuclear family. Since half the genetic variance occurs within families, estimates of individual genetic risk based on individual genomic profiles have the potential to be much more accurate predictors of individual genetic risk than family history.

Comparing genomic profiles to family history as predictors of genetic risk

The maximum AUC assuming complete knowledge of disease status of two parents was derived using equation 2 with family history scored as "yes" (at least one parent affected) or "no" (no parent affected): The probability of a parent chosen at random being affected is K, the probability an individual has at least one parent affected (family history "yes") is $2K - K^2$, assuming there is no assortative mating. Those without family history have equal rank of $(1 + (1\text{-}2K+K^2)N)/2$ since they take up rank positions of 1 to $((1\text{-}2K+K^2)N$, whereas those with family history have equal rank of $((1\text{-}2K+K^2)\text{N} + 1 + \text{N})/2$. Of those with "yes" family history we expect the proportion $2K_R - K_R^2 = 2\lambda_R K - (\lambda_R K)^2$ to be affected and proportion $(1 - 2\lambda_R K + (\lambda_R K)^2)$ not to be affected allowing us to calculate the mean rank of those affected, $\overline{r_d}$, and

$$AUC_{FH:2parent} = \frac{((2-\lambda_R K)\lambda_R + (K-3))K+1}{2(1-K)} \ . \qquad \text{(S1)}$$

This relationship makes no assumption about whether the whether the increased prevalence in the parents results from shared genetic or environmental risk factors. However, in order to provide an upper limit for AUC based on more extended family history we calculated AUC in a simulation using large average family size (Poisson distributed with mean 3 children per couple) and complete knowledge of disease status of relatives over three generations, assuming no family common environmental risk factors. A family history score was created weighting the proportion of relatives of each type who are affected by the genetic relationship to the individual for whom the genetic risk is predicted i.e., ½ for first degree relatives (parents, siblings), ¼ for second degree relatives (grandparents, avuncular) and ⅛ for third degree relatives (cousins).

AUC from family history

The AUC from classification on family history of both parents calculated from equation (S1) agreed well with that from simulation ±0.01 (results not presented). The AUC based on full knowledge of three generation family history from simulation is presented in Table S1, representing an upper limit of the predictive value of family history (resulting from shared genetic risk factors only). The maximum AUC for family history even under the most optimistic scenario is always less than 0.75 (and often considerably so) and accounts for only a small proportion (maximum of 0.25 for the diseases listed) of the known genetic variance.

References

1. Murff HJ, Spigel DR, Syngal S (2004) Does this patient have a family history of cancer? An evidence-based analysis of the accuracy of family cancer history. Jama-Journal of the American Medical Association 292: 1480-1489.
2. Scheuner MT, Whitworth WC, McGruder H, Yoon PW, Khoury MJ (2006) Expanding the definition of a positive family history for early-onset coronary heart disease. Genetics in Medicine 8: 491-501.
3. Feng R, McClure LA, Tiwari HK, Howard G (2009) A new estimate of family disease history providing improved prediction of disease risks. Statistics in Medicine 28: 1269-1283.
4. Eaves LJ, Kendler KS, Schulz SC, 2nd (1986) The familial sporadic classification: its power for the resolution of genetic and environmental etiologic factors. J Psychiatr Res 20: 115-130.
5. Kendler KS (1987) Sporadic vs familial classification given etiologic heterogeneity: I. Sensitivity, specificity, and positive and negative predictive value. Genet Epidemiol 4: 313-330.
6. Yang J, Visscher PM, Wray NR (2009) Sporadic cases are the norm for common disease. European Journal of Human Genetics 2009 Oct 14. [Epub ahead of print].
